# Efficacy and Safety of Coenzyme Q10 Supplementation in Neonates, Infants and Children: An Overview

**DOI:** 10.3390/antiox13050530

**Published:** 2024-04-26

**Authors:** David Mantle, Iain Parry Hargreaves

**Affiliations:** 1 Pharma Nord (UK) Ltd., Morpeth, Northumberland NE61 2DB, UK; 2 School of Pharmacy and Biomolecular Sciences, Liverpool John Moores University, Merseyside L3 5UX, UK; I.hargreaves@ucl.ac.uk

**Keywords:** coenzyme Q10, ubiquinone, primary CoQ10 deficiency, acyl CoA dehydrogenase deficiency, Duchenne muscular dystrophy, migraine, Down syndrome, ADHD, idiopathic cardiomyopathy, Friedreich’s ataxia

## Abstract

To date, there have been no review articles specifically relating to the general efficacy and safety of coenzyme Q10 (CoQ10) supplementation in younger subjects. In this article, we therefore reviewed the efficacy and safety of CoQ10 supplementation in neonates (less than 1 month of age), infants (up to 1 year of age) and children (up to 12 years of age). As there is no rationale for the supplementation of CoQ10 in normal younger subjects (as there is in otherwise healthy older subjects), all of the articles in the medical literature reviewed in the present article therefore refer to the supplementation of CoQ10 in younger subjects with a variety of clinical disorders; these include primary CoQ10 deficiency, acyl CoA dehydrogenase deficiency, Duchenne muscular dystrophy, migraine, Down syndrome, ADHD, idiopathic cardiomyopathy and Friedreich’s ataxia.

## 1. Introduction

Coenzyme Q10 (CoQ10) is usually described as a vitamin-like substance, although by definition, CoQ10 is not a vitamin since it is produced by various tissues within the human body. CoQ10 comprises a central benzoquinone ring bound to a polyisoprenoid side chain composed of ten isoprene units. Based on this structure, CoQ10 is one of the most lipophilic naturally occurring substances, and as such, is located within intracellular membranes, principally in mitochondria, but also within the membranes of other subcellular organelles such as lysosomes, peroxisomes and Golgi apparatus. The benzoquinone ring contains redox active sites, whereas the polyisoprenoid chain is responsible for positioning the CoQ10 molecule within the mid-plane of the lipid bilayer of various cell membrane types [1]. CoQ10 has a key role in many aspects of cellular metabolism; in addition to its role in the generation of ATP via oxidative phosphorylation within mitochondria, it is an important antioxidant; has roles in the metabolism of fatty acids, amino acids, sulphides and cholesterol; and has been shown to directly affect the expression of a number of genes, including those involved in inflammation. CoQ10 exists in both oxidised (ubiquinone) and reduced (ubiquinol) forms, and for CoQ10 to function normally in the above intracellular processes, continual inter-conversion between these two forms is necessary [1]. The daily requirement for CoQ10 is not known with certainty but has been estimated to be approximately 500 mg/day, based on a total body pool of 2000 mg and an average tissue turnover time of four days. A small amount of CoQ10 (approximately 5 mg) is obtained from the daily diet, with most of the daily requirements being synthesised within the body. Deficiency of CoQ10 is broadly divided into primary CoQ10 deficiency and secondary CoQ10 deficiency. Primary CoQ10 deficiency results from mutations in genes involved in the CoQ10 biosynthetic pathway. The biosynthesis of CoQ is a complex multi-step process that takes place in various sub-cellular locations. At least 10 genes are required for the biosynthesis of functional CoQ10, a mutation in any one of which can result in a deficit in CoQ10 status [2]. Secondary coenzyme Q10 deficiency results from mutations in genes that are not directly related to the CoQ10 biosynthetic pathway or to non-genetic factors associated with various disorders [3]. The relevance of both primary and secondary CoQ10 deficiency in younger subjects is discussed in subsequent sections of this review.

To date, there appear to be no review articles specifically relating to the efficacy and safety of CoQ10 supplementation in younger subjects (as listed on Medline). Optimum endogenous CoQ10 synthesis takes place up to the age of 25 years [4], so there is no rationale for the supplementation of CoQ10 in normal younger subjects (as there is in otherwise healthy older subjects). All of the articles in the medical literature, therefore, refer to the supplementation of CoQ10 in younger subjects with a variety of clinical disorders. In this article, we reviewed the efficacy and safety of CoQ10 supplementation in neonates (less than 1 month of age), infants (up to 1 year of age) and children (up to 12 years of age) in such subjects.

Clinical studies supplementing CoQ10 in neonates and infants have been restricted to subjects with primary CoQ10 deficiency and acyl CoA dehydrogenase deficiency, respectively. With regard to children, clinical studies supplementing CoQ10 are much broader in scope in terms of the number of studies, the number of patients and the types of disorders studied. The purpose of this review is as follows: (i) to provide a brief background to the diverse types of disorders covered by this article; (ii) to provide information relating to normal ranges for CoQ10 tissue levels in the respective age groups; (iii) to review the efficacy and safety of CoQ10 supplementation in clinical studies of various disorders in the different age groups.

## 2. Background to Disorders Covered by This Review

**Primary CoQ10 deficiency** results from mutations in genes involved in the CoQ10 biosynthetic pathway. In humans, at least 10 genes are required for the biosynthesis of functional CoQ10, a mutation in any one of which can result in a deficit in CoQ10 status. Primary CoQ10 deficiency is a very rare disorder, the prevalence and incidence of which have been estimated to be less than 1 per 100,000 population, respectively. Primary CoQ10 deficiency resulting from COQ gene mutations is associated with a heterogeneous spectrum of clinical phenotypes; however, the following generalisations may be made: (1) the disorder typically has a neonatal, infantile or childhood age of onset; (2) patients typically present with neurological dysfunction (encephalopathy, psychomotor delay, cerebellar atrophy/ataxia, optic atrophy), nephropathy, cardiomyopathy or any combination thereof; (3) the outcome for patients is typically either serious disability or fatality [2].

The only treatment for primary CoQ10 deficiency is oral supplementation with CoQ10, to which patients may respond favourably. However, the condition must be recognised sufficiently early since once damage to critical organs such as the kidney or the nervous system is established, only minimal recovery is possible. In general terms (i.e., all age groups), some forty clinical studies supplementing CoQ10 supplementation in primary CoQ10 deficiency have been reported in the medical literature; some 300 patients with primary CoQ10 deficiency have been identified, in approximately 100 of whom CoQ10 supplementation was attempted [2].

It should be noted that because of the rarity of the disorder, no formal clinical trials (randomised controlled or otherwise) have been reported in the medical literature. There is no consensus as to the most suitable dose of CoQ10, but doses used in clinical studies are typically in the range of 10–30 mg/kg/day for varying time periods from several months upwards [2]. The response to therapy depends on which COQ gene has been affected, the particular location of the mutation within each of the respective genes and the tissue(s) affected [2]. Steroid-resistant nephrotic syndrome resulting from COQ mutations appears to be particularly amenable to treatment via CoQ10 supplementation [2]. Clinical studies relating to CoQ10 supplementation in patients with primary CoQ10 deficiency have been summarised for neonates, infants and children in Table 1, Table 2 and Table 3, respectively; further details of individual studies are given in the review by Mantle et al. [2].

**Acyl-CoA dehydrogenase deficiency** is an inherited (autosomal recessive) disorder of mitochondrial fatty acid beta-oxidation. This disorder results from a defect of electron transport from FAD-containing CoA dehydrogenases to CoQ10 in the mitochondrial electron transport chain; this, in turn, is due to mutations in one of the three genes, two of which encode the alpha- and beta-subunits of the electron transfer flavoprotein, while the third encodes ETF ubiquinone oxidoreductase. This disorder is associated with mitochondrial dysfunction, oxidative stress, and inflammation. Symptoms typically include hypoglycaemia, muscle weakness and fatigue.

Acyl-CoA dehydrogenase deficiency is divided into type I (neonatal onset with congenital anomalies), type II (neonatal onset without congenital anomalies) and type III (late onset) forms. Individuals with type I or II forms typically present in the neonatal period and may die despite treatment [5]. Patients may have depleted levels of CoQ10; cultured fibroblasts from patients with acyl-CoA dehydrogenase deficiency had depleted levels of CoQ10 and increased levels of oxidative stress. Supplementation with CoQ10 increased cellular CoQ10 levels and decreased oxidative stress [6]. Depleted levels of CoQ10 have been reported in muscle biopsies from patients with acyl-CoA dehydrogenase deficiency [7,8], although not all patients are deficient in CoQ10 [9]. In general terms, supplementation with a combination of CoQ10 (60–240 mg/day), riboflavin (a precursor in the synthesis of FAD, 100–300 mg/day) and L-carnitine (50–100 mg/day) may result in significant symptomatic improvement.

**Duchenne muscular dystrophy (DMD)** is an X-linked recessive myopathy resulting from mutations in the gene encoding the membrane-associated protein dystrophin. DMD is the most severe form of muscular dystrophy, characterised by progressive wasting of skeletal muscles followed by death in the second or third decades of life due to failure of the cardiac and respiratory musculature. The incidence of DMD is estimated at 1 per 3500 live male births [10].

**Migraine** is the most common type of headache diagnosed in children. Migraine causes school absenteeism, restrictions on home activities and restrictions on social activities, and has a significant impact on patient lives. There is evidence that mitochondrial dysfunction and oxidative stress may be involved in the pathogenesis of migraine, resulting in defective energy metabolism, in turn impacting neurovascular function [11]. A number of clinical studies have indicated that CoQ10 supplementation in adults is effective in relieving some of the symptoms of migraine [12,13], but there is less clinical evidence supporting the use of CoQ10 in children. 

**Down syndrome** is a neurological disorder resulting from the presence of an additional copy of chromosome 21. In addition to intellectual disability, Down syndrome patients have an increased risk of pulmonary and cardiac disease [14]. There is evidence for mitochondrial dysfunction and oxidative stress in Down syndrome [15]. The incidence of Down syndrome is estimated as 1 per 1000 births.

**Attention deficit hyperactivity disorder (ADHD)** is a chronic neurobehavioral disorder characterised by inattention and/or hyperactivity–impulsivity associated with sleep problems in children [16]. Autism spectrum disorder (ASD) is a neurodevelopmental disorder characterised by early-onset impairments in social communication and interaction, as well as by restricted, repetitive interests and behaviour [17]. There is evidence for the involvement of mitochondrial dysfunction and oxidative stress in the pathogenesis of both ADHD and ASD [18,19,20].

**Idiopathic cardiomyopathy:** Although cardiomyopathy may result from primary CoQ10 deficiency, most cases of cardiomyopathy in infants or children are of idiopathic aetiology; the patient outcome is generally poor, often resulting in death within 2 years [21].

**Friedreich’s ataxia** is an autosomal recessive spinocerebellar ataxia resulting from mutations in the FXN gene, which encodes the protein frataxin [22]. The latter protein is involved in the regulation of intracellular iron metabolism, and frataxin deficiency results in mitochondrial iron deposition, increased oxidative stress, mitochondrial dysfunction and impaired cellular energy generation [23]. In addition to neurological dysfunction, Friedreich’s ataxia is strongly associated with cardiomyopathy [24].

**Table 1:** Clinical studies supplementing CoQ10 in neonates with primary CoQ10 deficiency.

Study/Number of Patients	Mutation Type/Presentation	Age at Presentation/Supplementation	Outcome
Scalais et al. [25]/1	COQ2/myoclonic seizures, cardiomyopathy	3 weeks/2 months	Died at 5 months after 5–30 mg/kg/day
Eroglu et al. [26]/3	COQ2/		
patient 1	proteinuria	4 days/3 months	Died at 4 months after 30 mg/kg/day
patient 2	neonatal diabetes	1 day/1 day	Died at 30 months after 30 mg/kg/day
patient 3	proteinuria	5 days/5 days	Died at 14 months after 30 mg/kg/day
Yu et al. [27]/5	COQ4/		
patient 1	metabolic acidosis	7 days/5 months	Died at 8 months after 40 mg/kg/day
patient 2	metabolic acidosis	1 day/2 days	Died at 3 days after 100 mg/kg/day
patient 3	cardiac failure	22 days/22 days	Cardiac function normalised at 32 days; dose not specified
patient 4	metabolic acidosis	1 day/4 years 5 months	Less responsive to supplementation; dose not specified
patient 5	metabolic acidosis	<1 month/1 year	Died at 13 months; dose not specified
Kwong et al. [28]/1	COQ7/heart and respiratory failure	4 days/2 months	Died at 1 year after 20–30 mg/kg/day
Duncan et al. [29]/1	COQ9/seizures	6 h/11.5 months	Died at 2 years after 300 mg/day

**Table 2:** Clinical studies supplementing CoQ10 in infants with primary CoQ10 deficiency.

Study/Number of Patients	Mutation Type/Presentation	Age at Presentation/ Supplementation	Outcome of CoQ10 Supplementation
Bellusci et al. [30]/1	COQ1 (PDSS2)/ Numerous morbidities	5 months/5 months	15–30 mg/kg/day. No effect on disease progression; died at 3 years.
Lopez et al. [31]/1	COQ1 (PDSS2)/ Nephrotic syndrome and encephalopathy	3 months/3 months	50 mg/day. No effect on disease progression; died at 8 months
Xu et al. [32]/1	COQ2/ nephrotic syndrome	11 months/13 months	30 mg/kg/day improved renal function
Starr et al. [33]/1	COQ2/ nephrotic syndrome	9 months/10 months	50 mg/kg/day improved renal function
Li et al. [34]/1	COQ2/proteinuria	7 months/11 months	30 mg/kg/day reduced proteinuria
Drovandi et al. [35]/32	COQ2/nephrotic syndrome	8 months/2.6 years	Up to 60 mg/kg/day improved proteinuria
Lu et al. [36]/1	COQ4/Leigh syndrome	12 months/12 months	Patient stabilised after 50 mg/kg/day
Yu et al. [27]/3	COQ4/		
patient 1	metabolic acidosis, dystonia	5 months/2 years	no improvement after supplementation (dose not specified), died at 3 years 6 months
patient 2	metabolic acidosis, spasms	6 months/9 months	improved responsiveness (dose not specified)
patient 3	seizures	2 months/11 months	Seizure control improved after 30 mg/kg/day
Cao et al. [37]/1	COQ6/nephrotic syndrome	10 months/10 months	Normal renal function restored after 30 mg/kg/day for 3 months
Heeringa et al. [38]/1	COQ6/nephrotic syndrome	2 months/2 months	Proteinuria improved after 15 mg/kg/day
Ashraf et al. [39]/1	COQ8B/nephrotic syndrome	<12 months/12 months	Partial remission of nephropathy after 100 mg/day
Feng et al. [40]/1	COQ8B/seizures	9 months/9 months	Proteinuria showed good response after 15–30 mg/kg/day
Olgac et al. [41]/1	COQ9/seizures	9 months/10 months	No neurological improvement after 5–50 mg/kg/day

**Table 3:** Clinical studies supplementing CoQ10 in children with primary CoQ10 deficiency.

Study/Number of Patients	Mutation Type/Presentation	Age at Presentation/Supplementation	Outcome of CoQ10 Supplementation
Diomedi-Camassei et al. [42]/1	COQ2/nephrotic syndrome	18 months/18 months	Clinical stabilisation after 30 mg/kg/day
Starr et al. [33]/2	COQ2/ nephrotic syndrome	(1) 10 years/10 years 8 months (2) 2 years/2 years 9 months	(1) No improvement after 50 mg/kg/day (2) Improved renal function after 50 mg/kg/day
Salviati et al. [43]/1	COQ4/neuromuscular symptoms	3 years/3 years	Significant symptomatic improvement after 30 mg/kg/day
Bosch et al. [44]/2	COQ4/spinocerebellar ataxia and stroke-like episodes	(1) 4 years/13 years (2) 9 years/11 years	No improvement in either patient after 1000 mg/day
Caglayan et al. [45]/1	COQ4/ataxia	8 years/26 years	Ataxia improved after 2000 mg/day
Malicdan et al. [46]/3	COQ5/ataxia, developmental delay	Early childhood/twenty years	Improved ataxia/cognitive function after 15 mg/kg/day ubiquinol
Heeringa et al. [38]/1	COQ6/nephrotic syndrome	2.5 years/5.5 years	Proteinuria improved after 15 mg/kg/day
Stanczyk et al. [47]/1	COQ6/nephropathy	2 years/4 years	Complete remission after 30 mg/kg/day
Nam et al. [48]/12	COQ6/nephropathy, hearing loss	2.7 years (mean)/5.6 years (mean)	Hearing loss in 50% of patients responded well after 30 mg/kg CoQ10
Mollet et al. [49]/3	COQ8A/ (1) cerebellar ataxia (2) developmental delay, seizures, ataxia (3) as for patient 2	(1) 18 months/3 years 8 months (2) 2 years/14 years (3) as for patient 2	No improvement in any of 3 patients following 10–20 mg/kg/day
Blumkin et al. [50]/1	COQ8A/cerebellar ataxia	2 years/5 years	Improved motor and cognitive abilities after 20 mg/kg/day
Jacobsen et al. [51]/2	COQ8A/ataxia, cerebellar atrophy	26–30 months/not specified	Ataxia improved after 20 mg/kg/day
Ucella et al. [52]/1	COQ8A/ataxia, seizures	8 years 6 months/14 years	Disease progression slowed after 15 mg/kg/day
Paprocka et al. [53]/1	COQ8A/developmental regression	22 months/22 months	Improved communication after 300 mg/day
Atmaca et al. [54]/8	COQ8B/proteinuria	16.8 years/18.9 years	Improved proteinuria after 30 mg/kg/day
Feng et al. [40]/1	COQ8B/nephrotic syndrome	9 years/11 years	No improvement after 30 mg/kg/day
Song et al. [55]/20	COQ8B/nephrotic syndrome	5–9 years/not specified	Reduced proteinuria in trial group of 5 subjects after 15–30 mg/kg/day

## 3. CoQ10 Normal Ranges in Neonates, Infants and Children

A number of studies have measured total (ubiquinone plus ubiquinol) CoQ10 levels in plasma or skeletal muscle samples in younger normal control subjects (the following values are given in terms of range or mean +/− SD as indicated). With regard to plasma CoQ10 levels, in a series of ten healthy neonates, Finckh et al. [56] measured plasma CoQ10 levels over the first 6 days of life; mean plasma CoQ10 levels over this time period were in the range of 0.51 +/− 0.10 to 0.63 +/− 0.05 umol/L. Niklowitz et al. [57] determined plasma CoQ10 levels in 140 children (age range 0.8–15.3 years) divided into 2-year increments; values (umol/L) ranged from 0.81 +/− 0.22 to 0.90 +/− 0.27, with no significant difference between the various 2-year age increments. Artuch et al. [58] divided their subjects into younger (1 month–7 years, n = 62) and older (8–18 years, n = 40) age groups; mean plasma CoQ10 levels were 0.80 umol/L (range 0.46–1.38 umol/L) and 0.57 umol/L (range 0.34–1.03 umol/L), respectively. Miles et al. [59] compared levels in 50 younger children (mean age 3.3 years, range 0.2–7.6 years) and 18 older children (mean age 13.8 years, range 10.4–17.4 years); mean plasma levels (umol/L) in these groups were 1.06 +/− 0.32 and 0.88 +/− 0.26, respectively, compared to a value of 1.04 +/− 0.33 in adults. In studies in which subject ages had not been stratified, Wittenstein et al. [60] quoted a mean plasma CoQ10 level of 0.68 umol/L (range 0.24–1.82 umol/L) in a series of 20 normal children with a mean age of 8 years (range 1–19 years). In a series of 50 children (mean age 12.6 years, range 10.9–13.3 years), Menke et al. [61] reported a mean plasma CoQ10 level of 0.79 umol/L (range 0.62–0.95 umol/L). In a series of 50 healthy children aged 2 months to 15 years, Becker et al. [62] quoted a mean plasma CoQ10 level of 0.87 +/− 0.31 umol/L.

With regard to skeletal muscle, in children (mean age 7.0 +/− 5.2 years), Miles et al. [63] quoted a normal value for total CoQ10 of 215 +/− 48.8 nmol/gm protein for biopsied quadriceps muscle. In a series of 140 normal children (mean age 4.7 years, range 0.8–15 years divided into 2-year increments), Niklowitz et al. [64] give a normal value for total CoQ10 in internal oblique muscle biopsies of 740 +/− 305 to 921 +/− 489 pmol/mg protein, with no significant difference between the various 2-year age increments. In a series of 80 normal children (mean age 7.2 years, range 0.2–18 years), a normal range for total CoQ10 in muscle biopsies (muscle types not specified) was given as 21.7–88.8 umol/g tissue [65].

## 4. CoQ10 Supplementation in Neonates

As noted above, clinical studies supplementing CoQ10 in neonates are restricted to subjects with primary CoQ10 deficiency or acyl-CoA dehydrogenase deficiency, respectively. With regard to primary CoQ10 deficiency, six studies have been reported in which CoQ10 supplementation was attempted; details of these studies are summarised in Table 1. The outcomes of many of these studies were unsuccessful in that the patients subsequently died immediately or subsequently over the course of 5–6 months or 1–2 years. There are a number of reasons why these studies reported no benefit of supplementation.

Of the clinical studies identified in this article in which supplemental CoQ10 had been used to treat patients with primary CoQ10 deficiency, only two studies specified the manufacturer of the supplement used. On this basis, it is difficult to judge the outcome of such studies in which poor-quality supplements may have been used. In contrast to prescription-type drugs, which require marketing authorisation by regulatory authorities, products classed as food supplements are not subject to the same regulatory standards. Thus, there is no regulatory requirement for the manufacturers of CoQ10 supplements to guarantee the quality, efficacy and safety of their products. In addition, in patients affected by neurological dysfunction, there is a question of whether supplemental CoQ10 is able to cross the blood–brain barrier; to date, there is no documented evidence that this occurs in human subjects.

In neonatal fatalities, there is a rationale for CoQ10 supplementation of at-risk mothers during pregnancy in order to reduce tissue damage during foetal development. However, it is well known that the placenta acts as a selective barrier between mother and foetus; whether supplemental CoQ10 can cross the placental barrier in humans has yet to be established and remains an issue for further research.

With regard to acyl-CoA dehydrogenase deficiency, only one case supplementing CoQ10 in neonates has been reported. Stanescu et al. [66] described a case with typical neonatal onset, with good longer-term outcomes (up to 4 years) following supplementation with riboflavin (300 mg/day) and CoQ10 (100 mg/day). It is of note that Yamada et al. [5] identified 15 patients with neonatal disease onset, all of whom had died within 2 years; because CoQ10 is not traditionally administered in Japan, CoQ10 supplementation was not attempted in any of the patients in this study. No studies were identified in which administration of supplemental CoQ10 to patients with acyl-CoA dehydrogenase deficiency resulted in adverse effects.

## 5. CoQ10 Supplementation in Infants

Clinical studies supplementing CoQ10 in infants (up to 1 year of age) were identified in children with primary CoQ10 deficiency (12 studies) and acyl-CoA dehydrogenase deficiency (three studies). Clinical studies supplementing CoQ10 in infants with primary CoQ10 deficiency are summarised in Table 2. The most consistent feature of these studies is the improvement in renal function in patients with steroid-resistant nephrotic syndrome following CoQ10 supplementation.

With regard to acyl-CoA dehydrogenase deficiency, Yuan et al. [67] described a 9-month-old infant with vomiting, muscle weakness, hypoglycaemia and glutaric aciduria resulting from mutation of the electron transfer flavoprotein (ETF)–ubiquinone oxidoreductase (ETF-QO) (*ETFDH*) gene. The patient was supplemented with CoQ10 (150 mg/day), vitamin B2 (150 mg/day) and L-carnitine; after one year, muscle strength and urinary organic acids were normalised, with the patient able to walk unaided. Kadoya et al. [68] reported an 8-month-old infant presenting with cardiomyopathy resulting from acyl-CoA dehydrogenase deficiency; the patient was subsequently supplemented with a combination of a beta-blocker, sodium pyruvate and CoQ10, resulting in normalisation of cardiac function. Saral et al. [69] described a case where hypoglycaemia and hypotonia were present immediately after birth but in which supplementation did not begin until the age of 8 months; supplementation with CoQ10 (100 mg/day, L-carnitine (50 mg/kg/day) and riboflavin (100 mg/day) resulted in normalisation of symptoms over the following two years.

## 6. CoQ10 Supplementation in Children

Clinical studies supplementing CoQ10 in children were identified in patients with primary CoQ10 deficiency (17 studies) and acyl-CoA dehydrogenase deficiency (1 study), together with a number of other disorders (13 studies). Eight studies were identified using the CoQ10 analogue idebenone. Clinical studies supplementing CoQ10 in children with primary CoQ10 deficiency are summarised in Table 3.

**Acyl CoA dehydrogenase deficiency:** Tummolo et al. [70] described an 11-year-old child presenting with vomiting and muscle weakness who was diagnosed with acyl CoA dehydrogenase deficiency; symptomatic improvement was reported after supplementation with a combination of CoQ10 (dose not specified), riboflavin and L-carnitine.

**Duchenne muscular dystrophy:** Several clinical studies have investigated the effect of CoQ10 supplementation in DMD. An open-label study by Spurney et al. [71] in a group of 12 DMD patients (age range 5–10 years) found that CoQ10 supplementation (90–500 mg/kg/day for 6 months) resulted in a mean 9% increase in muscle strength (assessed via the Quantitative Muscle Testing score), in addition to any steroid related improvement. However, the study was limited by the small cohort of patients, short duration of treatment, and lack of randomisation. No serious adverse effects were reported in this study.

Supplementation with the CoQ10 analogue idebenone reportedly benefits pulmonary function in DMD patients. A randomised controlled trial supplementing idebenone (900 mg/day for 12 months) in a series of 64 DMD patients (31 treated, 34 placebo; age range 10–18 years) significantly reduced loss of respiratory function (assessed via standard spirometry), as well as reducing administration of antibiotics for lung infections [72,73]. A follow-up study on a smaller group of 18 DMD patients confirmed the longer-term efficacy (up to 6 years) of CoQ10 supplementation in reducing the rate of decline in pulmonary function [74].

With regard to cardiac function, in an animal model of DMD, Buyse et al. [75] reported supplementation with CoQ10 (200 mg/kg/day) for up to 10 weeks in dystrophin-deficient mice significantly reduced inflammation and fibrosis in cardiac muscle, improved diastolic function and enhanced performance in exercise tolerance tests. However, a randomised controlled trial comprising 25 patients (aged 6–10 years; 12 treated and 13 placebo) with DMD found supplementation with CoQ10 (3–5 mg/kg/day for 6 months) had no significant benefit on echocardiographic parameters (as myocardial performance index) [76]; however, the small sample size was a major limitation of this study. Earlier studies by Folkers et al. [77] on patients with different types of muscular dystrophy, including Duchenne, Becker and limb-girdle dystrophies, reported improvements in cardiac function, as well as in skeletal muscle strength, following supplementation with CoQ10 (100 mg/day for 3 months).

**Migraine:** In a study of more than 1000 patients, CoQ10 levels were reported to be significantly reduced in some children and adolescents (mean age 13 years) suffering from migraines; supplementation of CoQ10 (1–3 mg/kg/day for 3 months) in those children with the lowest blood CoQ10 levels resulted in an improvement in headache frequency [78]. A randomised controlled trial comprising 72 patients (age range 5–15 years) suffering from migraine found supplementation with CoQ10 (30–60 mg/day for 3 months) resulted in a significant decrease in the frequency, duration and severity of attacks; these results were comparable to those obtained with amitriptyline, with fewer side effects [79]. Conversely, a randomised controlled trial comprising 120 children and adolescents supplemented with CoQ10 (100 mg/day for 7 months) found no significant difference in headache outcomes between treated and placebo groups at the end of the study, despite an initial symptomatic improvement over the first month [80].

**Down syndrome:** Oxidation of DNA has been implicated in the pathogenesis of Down syndrome, and blood CoQ10 levels are reportedly reduced in Down syndrome patients [81]. However, a randomised controlled trial supplementing CoQ10 (4 mg/kg/day) in Down syndrome patients had no significant effect on whole-body DNA oxidation (quantified via urinary excretion of 8-oxo-7,8-dihydro-2′-deoxyguanosine (8-oxodG) and 8-oxo-7,8-dihydroguanosine (8-oxoGuo), in both the short (6 months) and longer (4 years) [82,83].

**ADHD/autism:** Blood CoQ10 levels have been reported to be significantly reduced in children (aged 6–12 years) with ADHD compared to normal controls [84]. There has been one randomised controlled trial to date supplementing CoQ10; 60 children (ages 6–16) with ADHD were supplemented with CoQ10 (1–3 mg/kg/day for 6 months) and showed symptomatic improvement (particularly reduced hyperactivity), as well as reduced adverse effects from co-administered atomoxetine [85]. An open study of 90 children with autism supplemented with CoQ10 (30–60 mg/day for 3 months) showed reduced levels of blood oxidative stress markers and improved sleep patterns [86].

**Idiopathic cardiomyopathy:** An open study by Soongswang et al. [87] supplementing CoQ10 (3 mg/kg/day for 9 months) in 15 patients (mean age 4.4 years) reported improvement in some aspects of cardiac function/NYHA class. An open study by Chen et al. [88] supplementing CoQ10 (ubiquinol, 10 mg/kg/day for 6 months) in 10 children with idiopathic cardiomyopathy resulted in improved cardiac function (particularly ejection fraction)/NYHA class.

**Friedreich’s ataxia:** Contradictory outcomes have been described regarding the effect of supplementation with the CoQ10 analogue idebenone on cardiac function or neurological function in younger Friedreich’s ataxia patients. Thus, in an open study, supplementation with idebenone (5 mg/kg/day for 6 months) in eight children (age range 4–12 years) with Friedreich’s ataxia resulted in improved cardiac indices (left ventricular mass, left ventricular posterior wall thickness, shortening fraction) [89]. An open study of Friedreich’s ataxia patients, including 15 children (ages 4–12 years), found improved cardiac indices (left ventricular posterior wall thickness, interventricular septum thickness) following supplementation with idebenone (20 mg/kg/day) [90]. However, in a randomised controlled trial comprising 70 paediatric patients, supplementing idebenone (450–2250 mg/day for 6 months) did not result in any significant benefit on left ventricular hypertrophy or other cardiac function parameters [91]. 

Similarly, with regard to neurological function, an open study by Pineda et al. [92] comparing younger (ages 8–18 years) and adult (ages 18–46 years) patients with Friedreich’s ataxia found supplementation with idebenone (5–20 mg/kg/day for 3–5 years) stabilised neurological function in younger subjects, but not in adults. However, a randomised controlled trial supplementing idebenone (450–2250 mg/day for 6 months) in a series of 70 children/adolescents (aged 8–18 years) did not result in any significant improvement in neurological function [93]. In addition, a randomised controlled trial, supplementation with idebenone (5–45 mg/kg/day for 6 months) in a cohort of 42 children/adolescents (aged 9–17 years) with Friedreich’s ataxia did not result in any improvement in exercise capacity [94].

**Diabetes:** In an open study of 50 paediatric patients with type 1 diabetes, supplementation with CoQ10 (100 mg/day for 3 months) had no significant effect on fasting blood glucose or glycated haemoglobin levels; however, the treatment was reported to be well-tolerated [95].

## 7. Safety of CoQ10 Supplementation

In general terms, the safety of supplemental CoQ10 is well established. To date, more than 200 randomised controlled clinical trials are listed on Medline in which CoQ10 has been supplemented in a wide range of disorders; dosage regimes of the order of 200–300 mg/day for 3–6 months are typically utilised, although some studies have used much higher daily doses (2700 mg/day) or longer intervention periods (up to 5 years). None of these studies has reported any serious adverse effects as a result of CoQ10 supplementation. However, relatively few of these studies have involved younger subjects, and this issue is addressed in the following section.

As summarised in Table 1 and Table 2, in studies supplementing CoQ10 in neonatal or infantile patients with primary CoQ10 deficiency, a number of these subjects subsequently died, either immediately or subsequently over the course of 5–6 months or 1–2 years. However, this is a consequence of the disease process rather than the CoQ10 supplementation. No studies were identified in which serious adverse effects attributable to CoQ10 supplementation were reported. With regard to primary CoQ10 deficiency, in the study by Drovandi et al. [35] of 116 younger subjects, adverse effects associated with CoQ10 supplementation were reported as uncommon and mild; gastrointestinal disturbance was noted in five children, in two of whom supplementation was discontinued. Two of the studies listed in Table 2 [32,34] were in Chinese, for which no translations were available, and no reference to adverse effects resulting from CoQ10 supplementation was noted in the respective English language abstracts. None of the other studies listed in Table 2 or Table 3 reported any significant adverse effects attributable to CoQ10 supplementation.

The safety of CoQ10 supplementation in children with other types of disorders is now considered. In children with Duchenne muscular dystrophy, the study by Spurney et al. [54] reported no serious adverse events following CoQ10 supplementation; one patient developed a headache of moderate intensity associated with a dose of 400 mg/day, which was resolved on reduction in the dose to 200 mg/day. No adverse events were reported in the studies by Salehi et al. [76] and Folkers et al. [77] in children with muscular dystrophy.

In children with migraine, in the study by Yaghini et al. [79], one child was reported to suffer from abdominal discomfort following CoQ10 supplementation. No adverse effects were reported in the studies by Hershey et al. [78] or Slater et al. [80] following CoQ10 supplementation in children with migraine. No adverse effects resulting from long-term supplementation with CoQ10 (4 mg/kg/day for 4 years) were reported in children with Down syndrome [83]. In children with ADHD, adverse effects following CoQ10 supplementation were described in the study by Gamal et al. [85] as minimal. In children with type I diabetes, supplementation with CoQ10 was reported to be well tolerated [95].

Similarly to the scenario with CoQ10, no serious adverse events were reported in clinical studies supplementing the CoQ10 analogue idebenone. None of the studies supplementing idebenone in children with Friedreich’s ataxia reported any serious adverse events; the study by Hausse et al. [89] described increased appetite in some patients. In children with Duchenne muscular dystrophy, supplementation with idebenone reduced the number of bronchopulmonary adverse events (i.e., adverse events resulting from the disease process) compared to placebo [72].

## 8. Summary

**Figure 1:**
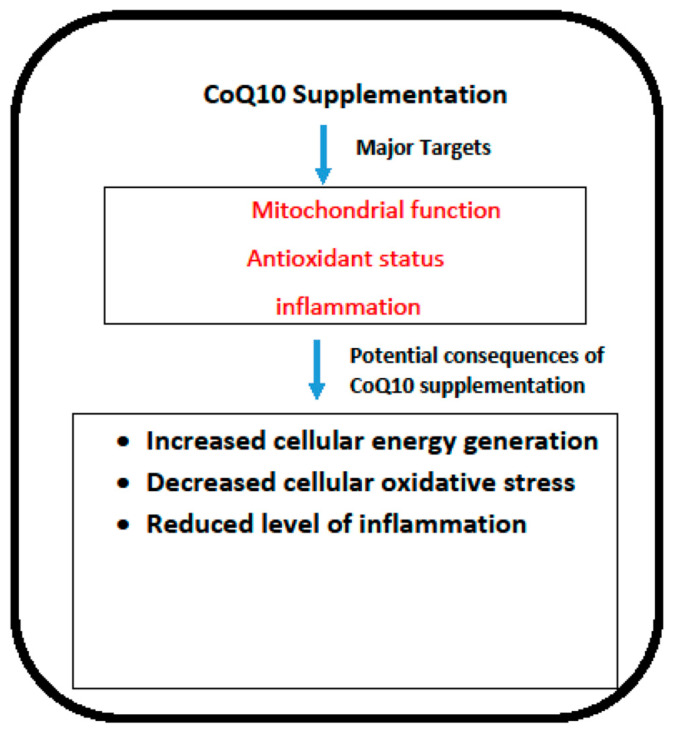
The potential targets and consequences of CoQ10 supplementation. COQ10: Coenzyme Q10.

In terms of efficacy, the most obvious application of CoQ10 supplementation is arguably in the treatment of primary CoQ10 deficiency. The major targets of CoQ10 supplementation are outlined in Figure 1. As is noted from the data summarised in this article, patients with this disorder may respond well to CoQ10 supplementation, although the response to therapy depends on which COQ gene has been affected, the particular location of the mutation within each of the respective genes, and the time interval between initial presentation and commencement of CoQ10 supplementation. The condition must be recognised sufficiently early since once damage to critical organs such as the kidney or the nervous system is established, only minimal recovery is possible. Steroid-resistant nephrotic syndrome resulting from COQ mutations appears to be particularly amenable to treatment via CoQ10 supplementation. It is of note that, at present, there is no consensus on the required plasma CoQ10 status required to elicit clinical improvement in patients. However, an in vitro study by Lopez et al. [96] and Duberley et al. [97] reported an improvement in bioenergetic status and normalisation of cellular oxidative stress in CoQ10 deficient fibroblasts and neuroblastoma cells following supplementation with 5 μM and 10 μM, respectively. These concentrations of CoQ10 may, therefore, be appropriate as targets for plasma levels in patients to achieve clinical improvement following CoQ10 supplementation. In neonatal fatalities, there is a rationale for CoQ10 supplementation of at-risk mothers during pregnancy in order to reduce tissue damage during foetal development. However, it is well known that the placenta acts as a selective barrier between mother and foetus; whether supplemental CoQ10 can cross the placental barrier in humans has yet to be established, and it remains an issue for further research.

Supplementation with CoQ10 or its analogue idebenone in younger subjects has also been reported to be of benefit in a number of other disorders, including acyl-CoA dehydrogenase deficiency, muscular dystrophy, migraine, ADHD, cardiomyopathy and Friedreich’s ataxia, as noted in the present article.

In general terms, the absorption of supplementary CoQ10, together with some aspects of subsequent metabolism, has been described in detail [98]. However, a number of issues relating to the metabolism of supplementary CoQ10 have still to be resolved: (i) whether the bioavailability of CoQ10 could be improved; (ii) whether CoQ10 could be administered intravenously; (iii) whether CoQ10 could be administered via alternative routes; (iv) whether CoQ10 can cross the blood–brain barrier or placental barrier; (v) how CoQ10 is transported into and within target cells; (vi) why some clinical trials supplementing CoQ10 may have been unsuccessful; and (vii) which is the most appropriate tissue for the clinical assessment of CoQ10 status [99]. These issues, whether in children or adults, remain subjects for future research. An additional issue for future research, perhaps more relevant to younger subjects, is whether the environment, in terms of geography or socioeconomic status, may affect CoQ10 status in the various age groups. With regard to safety, no evidence of any serious adverse events was identified in any of the studies reviewed in the present article; any adverse effects that occurred were rare and generally mild in nature. In more general terms, the safety of supplemental CoQ10 (in both animal models and humans) has been assessed by Hidaka et al. (2008) [100]; results from animal-based studies found supplemental CoQ10 to have low toxicity, with no evidence of effects on development or mutagenicity, and no evidence of the induction of serious adverse effects in humans. In addition, more than 200 randomised controlled clinical trials are currently listed on Medline in which supplementary CoQ10 has been administered in a variety of disorders, in various dosages (up to 3000 mg/day) and for various time periods (up to 5 years); in none of these studies were any serious adverse effects attributable to CoQ10 reported.

With regard to safety, no evidence of any serious adverse events was identified in any of the studies reviewed in the present article; any adverse effects that did occur were rare and generally mild in nature.

## Data Availability

Not applicable.
